# Effects of Short‐Term Energy Limitation at Different Levels With Normal Protein Intake on Hepatic Lipid Metabolism and the Gut Microbiota in Overweight/Obese Mice

**DOI:** 10.1002/fsn3.72362

**Published:** 2026-09-14

**Authors:** Ying Tian, Yao Tang, Mingxia Gu, Jiawei Gong, Ying Zhou, Yuqing He, Keyu Huang

**Affiliations:** ^1^ School of Public Health, Faculty of Medicine Yangzhou University Yangzhou China; ^2^ Department of Cardiology Nanjing Central Hospital Nanjing China

**Keywords:** energy limitation, gut microbiota, hepatic lipid metabolism, obesity, overweight, sex differences

## Abstract

This study investigated the sex‐specific effects of graded short‐term energy limitation (EL) with normal protein intake on hepatic lipid metabolism and the gut microbiota in overweight/obese mice. Mice were allocated to a normal control (NC) group, a high‐fat diet model (MC) group, and groups receiving 20%, 30% or 40% EL (*n* = 8 per group). All mice underwent blood biochemistry, liver biochemistry, histological, liver metabolomic, and fecal microbiota community genomic analyses. Relative to the NC group, both male and female MC mice developed varying degrees of insulin resistance, dyslipidaemia, and sex hormone dysregulation. However, disrupted hepatic lipid metabolism was detected solely in male mice, in association with changes in key lipid‐metabolizing enzymes and metabolites; female mice showed only disturbed total cholesterol (TC) metabolism, which was linked to alterations in the cholesterol synthesis rate‐limiting enzyme HMGCR. With normal protein intake, 20%, 30%, and 40% EL reduced hepatic triglyceride and TC synthesis in overweight/obese male mice by suppressing the expression of the key lipogenic enzyme DGAT and the activities of ACC and HMGCR (*p* < 0.05). An effect on the lipolytic enzymes CPT1 and CYP7A1 was detected only at 30% EL (*p* < 0.05), and hepatic metabolite profiles varied with the degree of EL. In female mice, only 40% EL significantly decreased TC synthesis by inhibiting both HMGCR expression and enzymatic activity (*p* < 0.05). Furthermore, irrespective of sex, short‐term EL (all levels) with normal protein intake reduced the gut Firmicutes/Bacteroidetes ratio in overweight/obese mice (*p* < 0.05). In conclusion, graded short‐term EL with adequate protein intake exerts differential effects on hepatic lipid metabolism and the gut microbiota in overweight/obese mice, with pronounced sex‐specific differences.

## Graphical abstract

These findings demonstrate that with adequate protein intake, graded short‐term EL (20%–40%) effectively improves hepatic lipid metabolism and modulates gut microbiota composition in overweight/obese mice, although these regulatory effects exhibit pronounced sex‐specific differences.

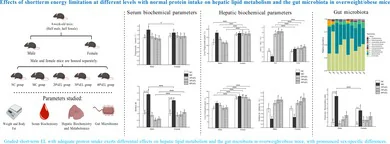

## 1 Introduction

The global obesity prevalence has doubled over the past four decades, imposing a substantial public health burden (Blüher 2019). Overweight/obesity is strongly linked to hepatic lipid dysregulation, a central pathological basis of metabolic syndrome, including insulin resistance and nonalcoholic fatty liver disease (Tacke et al. 2024). Therefore, ameliorating hepatic lipid dysregulation may alleviate hepatic steatosis and reverse metabolic syndrome through synergistic mechanisms.

Energy limitation (EL) is a classic non‐pharmacological intervention for hepatic lipid dysregulation in overweight/obesity (Kökten et al. 2021). The effect of EL on hepatic lipid metabolism varies with the degree of restriction (Gopalan et al. 2016; Wen et al. 2022; Zhang et al. 2022). However, existing studies have largely focused on single‐level EL regimens, leaving a considerable gap in the comparison of graded EL protocols and the elucidation of their mechanisms. The effects of EL on hepatic lipid metabolism are also sex‐dependent. We previously showed that 40% EL reduced hepatic fat content in both male and female overweight/obese rats but increased plasma total cholesterol only in males, with no change in females (Tian et al. 2025). Overall, how graded EL levels differentially affect hepatic lipid metabolism in overweight/obese mice—and the underlying mechanisms—remain poorly understood, and dynamic, high‐resolution metabolite‐level characterization is lacking.

The gut microbiota, often considered a “virtual organ”, regulates hepatic lipid metabolism through its metabolites via the gut–liver axis (Canfora et al. 2019). Diet is a key determinant of the intestinal environment, and different dietary patterns profoundly shape the composition, function, and metabolic activity of the gut microbiota (David et al. 2014). Obesity‐associated microbial dysbiosis further exacerbates hepatic lipid accumulation (Magne et al. 2020). EL can reshape the gut microbiota composition (Fan et al. 2023; van der Merwe et al. 2020). Moreover, sex differences may also influence EL‐mediated regulation of hepatic lipid metabolism by reshaping the gut microbiota (Li et al. 2021). However, most studies to date have not adequately considered sex or EL intensity as variables, focusing on a single sex or a single EL level; consequently, whether sex differences modulate the regulation of hepatic lipid metabolism, gut microbiota, and the metabolome across varying EL intensities remains unclear, severely limiting a comprehensive understanding of the mechanisms underlying EL intervention.

Collectively, how graded EL differentially affects hepatic lipid metabolism and the underlying mechanisms in overweight/obese mice remains unclear, particularly at the metabolite level. Dietary protein directly modulates hepatic de novo lipogenesis and fatty acid oxidation via amino acid sensing and gluconeogenesis, and indirectly regulates hepatic lipid homeostasis by remodeling the gut microbiota through the gut–liver axis (Delzenne et al. 2019; Liao et al. 2024). Therefore, in this study, we applied graded EL interventions with consistent protein intake in HFD‐induced overweight/obese mice, and used untargeted metabolomics and 16S rDNA sequencing to investigate the effects and mechanisms of different EL levels on hepatic TG/cholesterol metabolism and gut microbiota, including sex‐specific differences.

## 2 Methods

### 2.1 Animals and Diets

**FIGURE 1:**
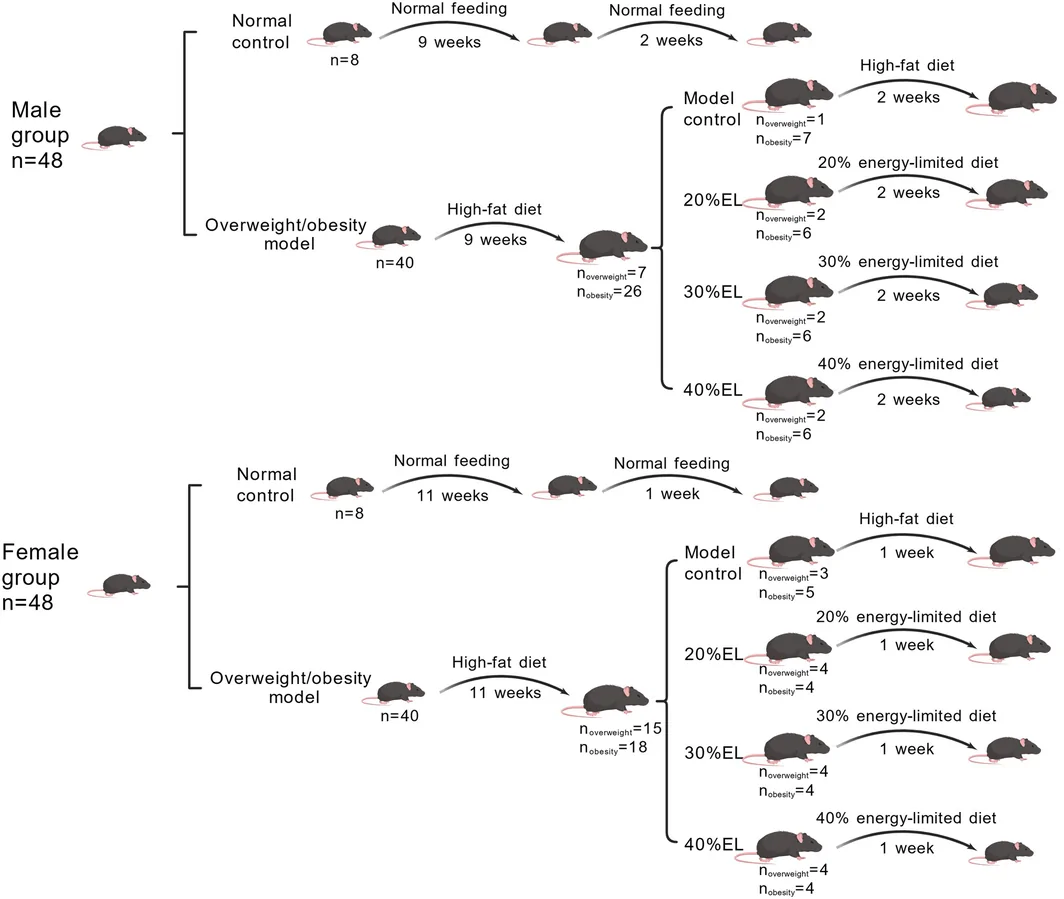
Experimental schedule.

We obtained 96 eight‐week‐old specific‐pathogen‐free C57BL/6J mice (48 male, 48 female) from the Institute of Comparative Medicine, Yangzhou University. Mice were singly housed with sexes separated under controlled conditions (22°C ± 2°C, 50% ± 10% relative humidity, 12‐h light/dark cycle). After 1 week of acclimatization, eight males and eight females were randomly assigned to the normal control (NC) group and received a standard maintenance diet (Standardization Administration of China 2010). The remaining mice received a high‐fat diet (HFD) to induce obesity. The model was considered established when the mean body weight exceeded that of the same‐sex NC group by 10%. Males reached this threshold after 9 weeks of HFD, whereas females required 11 weeks. Mice with body weights 10%–20% above the same‐sex NC group mean were classified as overweight, and those with body weights ≥ 20% above the mean as obese. Overweight/obese mice were then stratified by sex and randomly allocated to eight groups (*n* = 8 per group), such that initial body weights did not differ significantly among groups within each sex. These groups consisted of a male and a female MC group (continued HFD) and three male and three female EL intervention groups, which were fed the corresponding experimental diets (Figure 1). Detailed diet formulations, macronutrient composition and energy parameters are provided in Table S1.

The 20%, 30%, and 40% EL groups received 80%, 70%, and 60%, respectively, of the daily energy intake of the NC group. On day 1, the energy intake of the NC group was measured. From Day 2 onward, daily feed provision for each EL group was calculated according to Equation (1):

(1) $$I_{\mathrm{EL}}=\left[\left(1-N\right)\times I_{\mathrm{NC}}\times E_{\mathrm{NC}}\right]/E_{\mathrm{EL}}$$

where *I*_EL_ is the feed intake of the intervention group (g); *N* is the EL level (20%, 30% or 40%); *I*_NC_ is the previous day's feed intake of the NC group (g); *E*_NC_ is the energy density of the NC diet (kcal/100 g); *E*_EL_ is the energy density of the corresponding EL diet (kcal/100 g).

### 2.2 Sample Collection

Body weight was measured weekly for all groups. To prevent excessive weight loss and malnutrition from prolonged EL, the intervention was discontinued when the mean body weight of an EL group no longer differed significantly from that of the NC group (*p* > 0.05). Accordingly, the intervention was stopped at the end of Week 12 in males and Week 13 in females. After a 12‐h fast, mice were anesthetized with Zoletil, and blood was collected by cardiac puncture. Serum was obtained by low‐speed centrifugation. Following euthanasia by cervical dislocation, the liver and adipose depots (inguinal, peri‐epididymal or periovarian, and perirenal) were rapidly excised, rinsed with saline, blotted dry, and weighed. Fecal samples were collected from the distal colon adjacent to the anus and placed in 1.5‐mL sterile tubes. All samples were snap‐frozen in liquid nitrogen and stored at −80°C until analysis.

### 2.3 Serum Glycolipid Metabolism Parameters

Serum glucose (GLU), triglyceride (TG), total cholesterol (TC), low‐density lipoprotein (LDL), and high‐density lipoprotein (HDL) levels were measured with an automated clinical chemistry analyzer (Rayto Life Sciences, Shenzhen, China). Serum insulin (INS), glucagon (GC), estradiol (E2), and testosterone (T) concentrations were determined by ELISA kits (Shanghai Hengyuan Biological Technology, cat. nos. HB141‐Mu, HB128‐Mu, HS969‐Mu, and HS844‐Mu) according to the manufacturer's protocols. The homeostasis model assessment of insulin resistance (HOMA‐IR), the most widely used index derived from insulin measurements, was calculated using Equation (2) (Wallace et al. 2004):

(2) $$\begin{array}{cc} \text{HOMA}-\mathrm{IR}\;=\; & \left(\text{Fasting glucose}\;\left[\text{mmol}/L\right]\right.\\ & \left.\times \text{Fasting insulin}\;\left[\text{microU}/\mathrm{mL}\right]\right)/22.5 \end{array}$$

### 2.4 Measurement of Hepatic Lipid Metabolism‐Related Parameters

Frozen liver tissue was homogenized in isopropanol, sonicated, and centrifuged. Hepatic triglyceride (TG), total cholesterol (TC), acetyl‐CoA carboxylase (ACC), phosphorylated ACC (PACC), diacylglycerol acyltransferase (DGAT), carnitine palmitoyltransferase I (CPT1), HMG‐CoA reductase (HMGCR), phosphorylated HMGCR (PHMGCR), and cholesterol 7α‐hydroxylase (CYP7A1) were quantified in the supernatant using ELISA kits (Shanghai Hengyuan Biological Technology) according to the manufacturer's instructions. Catalog numbers: TG, HB1358‐Mu; TC, HB1371‐Mu; ACC, HB116‐Mu; PACC, HS1484‐Mu; DGAT, HB1987‐Mu; CPT1, HB437‐Mu; HMGCR, HB1858‐Mu; PHMGCR, HS1486‐Mu; CYP7A1, HB945‐Mu.

### 2.5 Liver Histology and Pathological Scoring

Liver tissues were fixed in 4% paraformaldehyde for 24 h, dehydrated through a graded ethanol series, cleared in xylene and embedded in paraffin. Serial 5‐μm sections were cut and stained with hematoxylin and eosin (H&E). Images were captured from five non‐overlapping fields per section at 200× magnification. Three pathologists blinded to group allocation independently scored the sections using the NAFLD activity score (NAS) system of Kleiner et al. (Kleiner et al. 2005) (steatosis, 0–3; lobular inflammation, 0–3; ballooning, 0–2). The mean total NAS (range 0–8) across the three pathologists was used for analysis. The percentage area of hepatic lipid vacuoles was quantified with ImageJ (National Institutes of Health). Briefly, ×200 field images were converted to 8‐bit greyscale, lipid vacuoles were segmented using a uniform threshold, and the area fraction was calculated. The mean of three fields per section was used for statistical analysis.

### 2.6 Untargeted Liver Metabolomics

#### 2.6.1 Data Acquisition

Chromatographic separation was carried out on a Vanquish UHPLC system (Thermo Scientific) using a Waters ACQUITY Premier HSS T3 column (100 × 2.1 mm, 1.8 μm) held at 40°C. Mobile phases were 0.1% formic acid in water (A) and 0.1% formic acid in acetonitrile (B). Gradient elution was used (programme in Table S2) at 0.4 mL/min. The injection volume was 4 μL. Metabolites were detected with a Q Exactive HF‐X high‐resolution mass spectrometer (Thermo Scientific) operated in both positive‐ and negative‐ion modes. Full‐scan MS data were collected over m/z 70–1050 at a resolution of 35,000 (AGC target, 1 × 10^6^; maximum injection time, 100 ms). Data‐dependent acquisition was applied to the three most intense ions, and MS/MS spectra were recorded at 17,500 resolution (AGC target, 1 × 10^5^; maximum injection time, 80 ms). A quality control (QC) sample, prepared by pooling equal volumes of all study samples, was injected every 10 samples to monitor system stability.

#### 2.6.2 Data Processing and Metabolite Identification

Raw mass spectrometry data were converted to mzXML format using ProteoWizard. Peak detection, retention‐time correction, and peak alignment were performed with the XCMS package in R. Metabolic features with missing values in > 50% of samples were removed; remaining missing values were imputed using the KNN algorithm. Batch and signal drift correction was subsequently performed using SVR based on the QC samples. After quality control filtering, principal component analysis (PCA) and orthogonal partial least‐squares discriminant analysis (OPLS‐DA) were performed. Model predictive ability was evaluated by sevenfold cross‐validation, and model reliability was assessed by 200 random permutation tests (*Q* ^2^ > 0.5 and *p* < 0.05). Significantly differential metabolites were screened using the criteria of variable importance in projection (VIP) > 1, FDR‐adjusted *p* < 0.05, and (|log_2_(fold change)| > 1), and were visualized in volcano plots. Structural identification was carried out by searching an in‐house library and public databases, such as HMDB and KEGG. Detailed information on all metabolites is provided in Appendix S1. To improve identification reliability, the metabolites retained after screening were further filtered through a secondary quality control requiring Level 1a structural confidence and a matching score > 0.8.

### 2.7 Gut Microbiota Analysis

Genomic DNA was extracted by the CTAB/SDS method (Zhou et al. 2020), verified by agarose gel electrophoresis, and diluted to 1 ng/μL. PCR amplification was performed using barcoded specific primers. PCR products were purified with magnetic beads, quantified, pooled, and recovered by gel extraction. Sequencing libraries were prepared with the TruSeq DNA PCR‐Free Kit, quantified by Qubit and qPCR, and sequenced on the NovaSeq6000 platform. Raw reads were merged with FLASH, and chimeras were detected and removed using vsearch. Amplicon sequence variants (ASVs) were generated by denoising with DADA2 and QIIME 2 (Bolyen et al. 2019). Taxonomic assignment was performed by aligning ASVs to the SILVA SSU rRNA database with mothur (confidence threshold 0.8–1), providing phylum‐level classification (Quast et al. 2013). Alpha diversity, Venn diagrams and principal coordinate analysis (PCoA) were computed in R. Community structure was visualized by PCoA on weighted UniFrac distances. Intergroup differences were assessed by global ADONIS and ANOSIM tests (9999 permutations); pairwise comparisons were performed with built‐in global correction. A significance threshold of *α* = 0.05 was applied. Phylum‐level taxonomic information is provided in Appendix S2.

### 2.8 Statistical Analysis

Longitudinal body weight data were analyzed using linear mixed‐effects models. The models included body weight as the dependent variable; group, week, and the group × week interaction were fixed effects. Mouse identity was fitted as a random intercept to account for the correlation among repeated measures within the same animal. When a significant group × week interaction was detected, simple‐effects tests were performed, with Bonferroni correction for multiple comparisons. A two‐sided α level of 0.05 was considered significant. Data are presented as mean ± standard error.

For non‐repeated measures, normality of model residuals was examined with the Shapiro–Wilk test and homogeneity of variances was assessed by Levene's test. When both assumptions were satisfied, a two‐way ANOVA was used to examine the main effects of sex and group and their interaction. When assumptions were violated, robust two‐way ANOVA on 20% trimmed means was performed, with robust pairwise comparisons for between‐group differences. The significance level was set at *α* = 0.05. When a significant sex × group interaction was observed, simple‐effects tests were conducted with Bonferroni correction. When the interaction was not significant, main effects were examined; significant main effects were followed by Tukey's HSD post hoc comparisons. Statistical analyses were conducted using SPSS 20 (IBM, Armonk, NY, USA) and R. Bar graphs were plotted with OriginPro 2025 (OriginLab, Northampton, MA, USA). Detailed results for all variables are provided in Table S3.

### 2.9 Ethics Statement

All experimental procedures and animal husbandry protocols were reviewed and approved by the Institutional Animal Care and Use Committee of Yangzhou University (approval No. 202407030). All animal work was performed in full compliance with the ARRIVE guidelines and the National Institutes of Health Guide for the Care and Use of Laboratory Animals.

## 3 Results

### 3.1 Body Weight, Adiposity, and Food Intake

**FIGURE 2:**
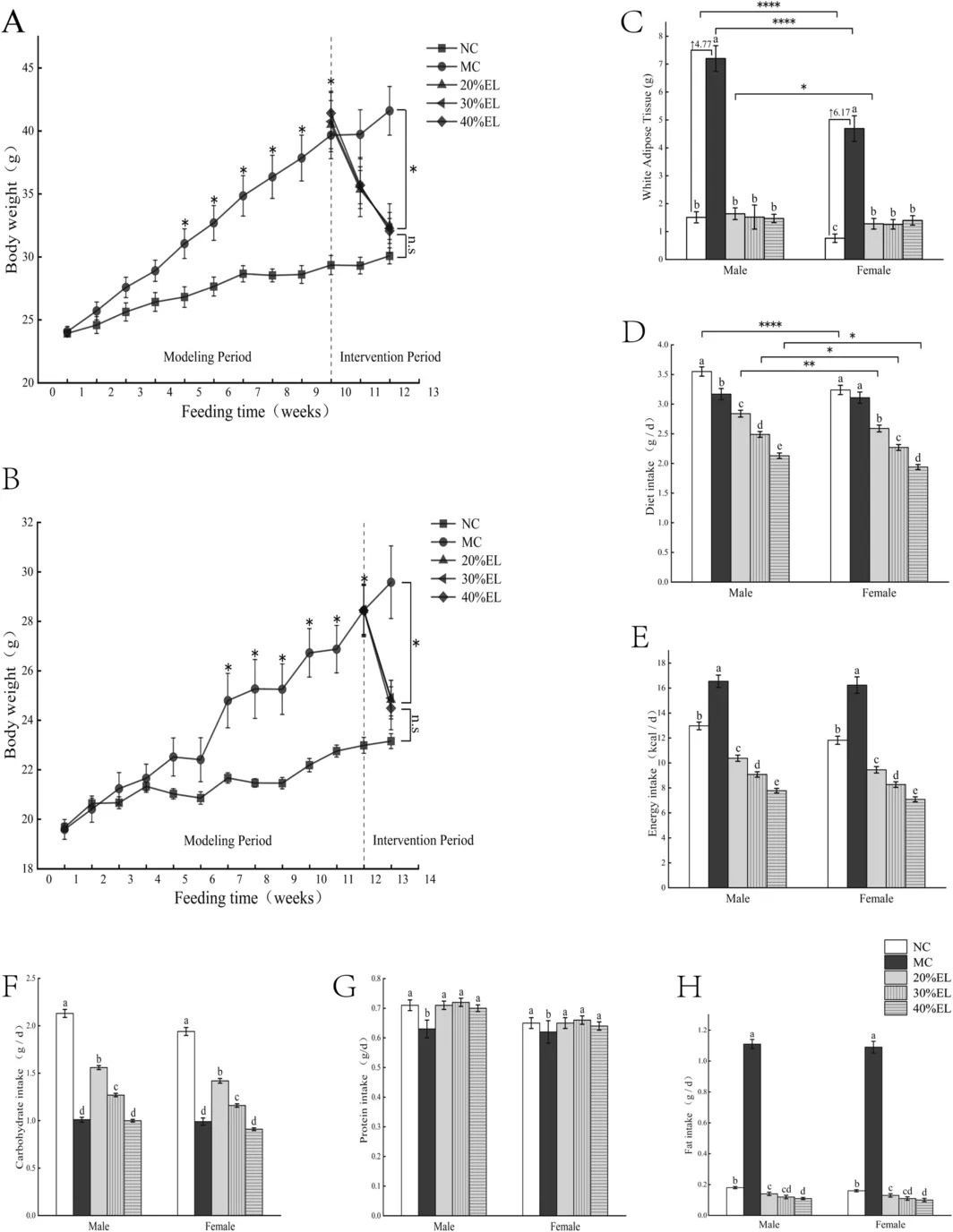
Body weight, white adipose tissue mass, and feed intake of mice. (A) Body weight changes in male mice. (B) Body weight changes in female mice. (C) White adipose tissue weight. (D) Feed intake. (E) Energy intake. (F) Carbohydrate intake; (G) Protein intake. (H) Fat intake. Data are presented as mean ± SEM (*n* = 8). Within the same sex, different letters indicate statistically significant differences among groups; asterisks denote significant differences between male and female mice under the same intervention: *p < 0.05; **p < 0.01; ***p < 0.001; ****p < 0.0001.

At baseline, initial body weights did not differ among groups within either sex (*p* > 0.05). After the induction of obesity, the MC group had significantly higher body weight than the NC group (*p* < 0.05). Following graded EL, body weights in both sexes decreased markedly and were comparable to those of the NC group (*p* > 0.05; Figure 2A,B). In the MC group, white adipose tissue weight was 4.77‐fold higher in males and 6.17‐fold higher in females than in the respective NC groups, indicating a more pronounced adipose accumulation in females in response to the high‐fat diet. After EL across all levels, white adipose tissue weight was significantly reduced in both sexes and no longer differed from that of the NC group (*p* > 0.05; Figure 2C). Figure 2D–H presents the energy and macronutrient intake levels of each group. Total energy and fat intakes were significantly higher in the MC group than in all other groups (*p* < 0.05). Carbohydrate intake in the NC group was significantly higher than that in the other groups (*p* < 0.05). No significant differences in protein intake were observed among the EL groups (*p* > 0.05).

### 3.2 Serum Biochemistry

**FIGURE 3:**
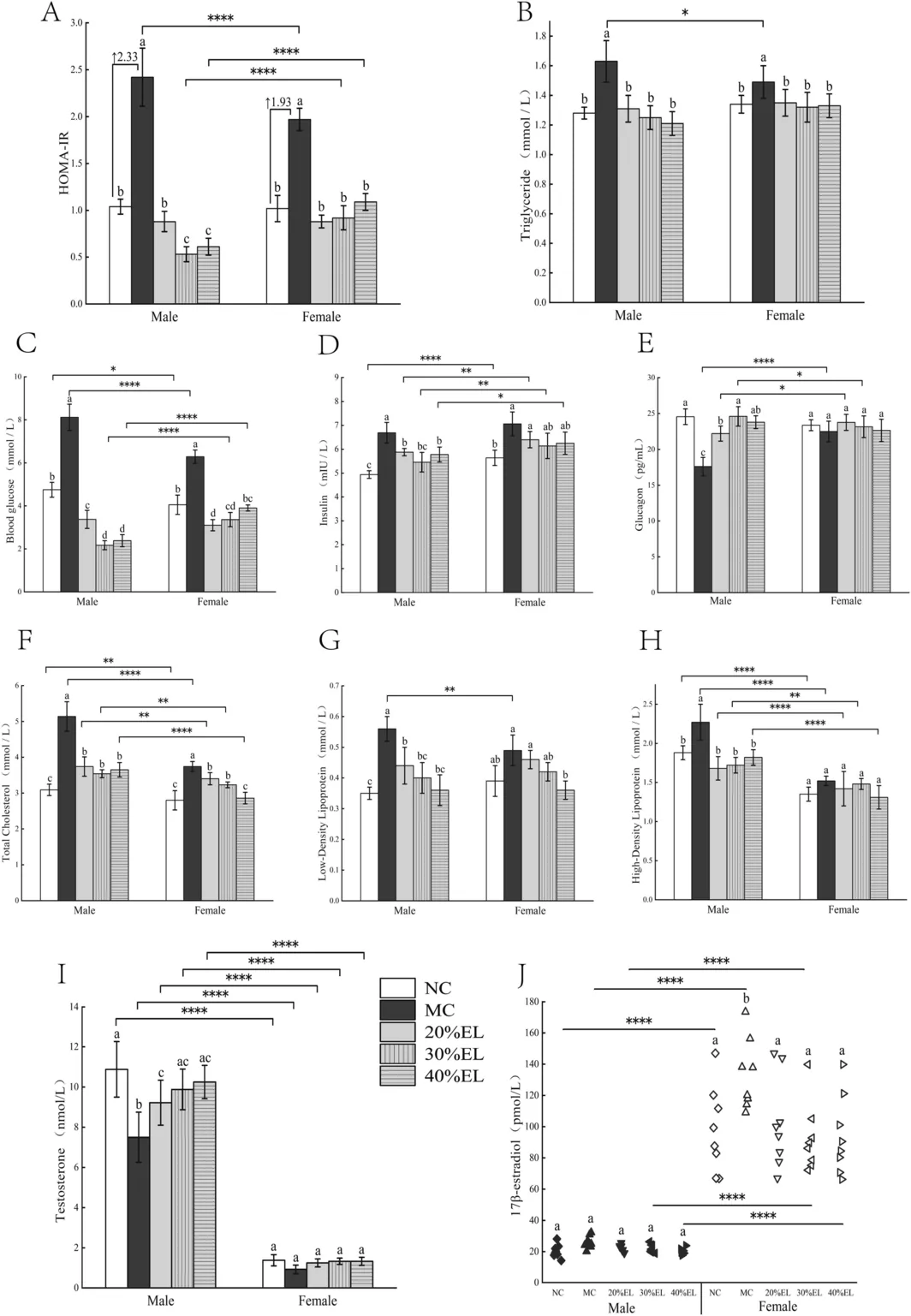
Serum glucose, lipid, and sex hormone levels in mice. (A) HOMA‐IR. (B) Serum TG content. (C) GLU content. (D) INS content. (E) GC content. (F) Serum TC content. (G) LDL content. (H) HDL content. (I) T content. (J) E2 content. Data are presented as mean ± SEM (*n* = 8). Within the same sex, different letters indicate statistically significant differences among groups; asterisks denote significant differences between male and female mice under the same intervention: *p < 0.05; **p < 0.01; ***p < 0.001; ****p < 0.0001.

Compared with the NC group, the MC group exhibited marked IR (*p* < 0.05): the HOMA‐IR index was 2.33‐fold higher in males and 1.93‐fold higher in females, and the sex difference was significant. All EL interventions significantly reversed IR in overweight/obese mice (Figure 3A,C–E). Serum TG, TC, and LDL levels were significantly elevated in the MC group relative to the NC group (*p* < 0.05). EL intervention improved these parameters overall; however, in female mice, TG, TC, and LDL were significantly reduced only after 40% EL (*p* < 0.05; Figure 3B,F–H). With respect to serum sex hormones (Figure 3I,J), male MC mice showed a marked decrease in T, whereas female MC mice displayed a significant increase in E2. Both 30% EL and 40% EL restored hormone levels to values comparable to those of the NC group (*p* < 0.05).

### 3.3 Liver Histology

**FIGURE 4:**
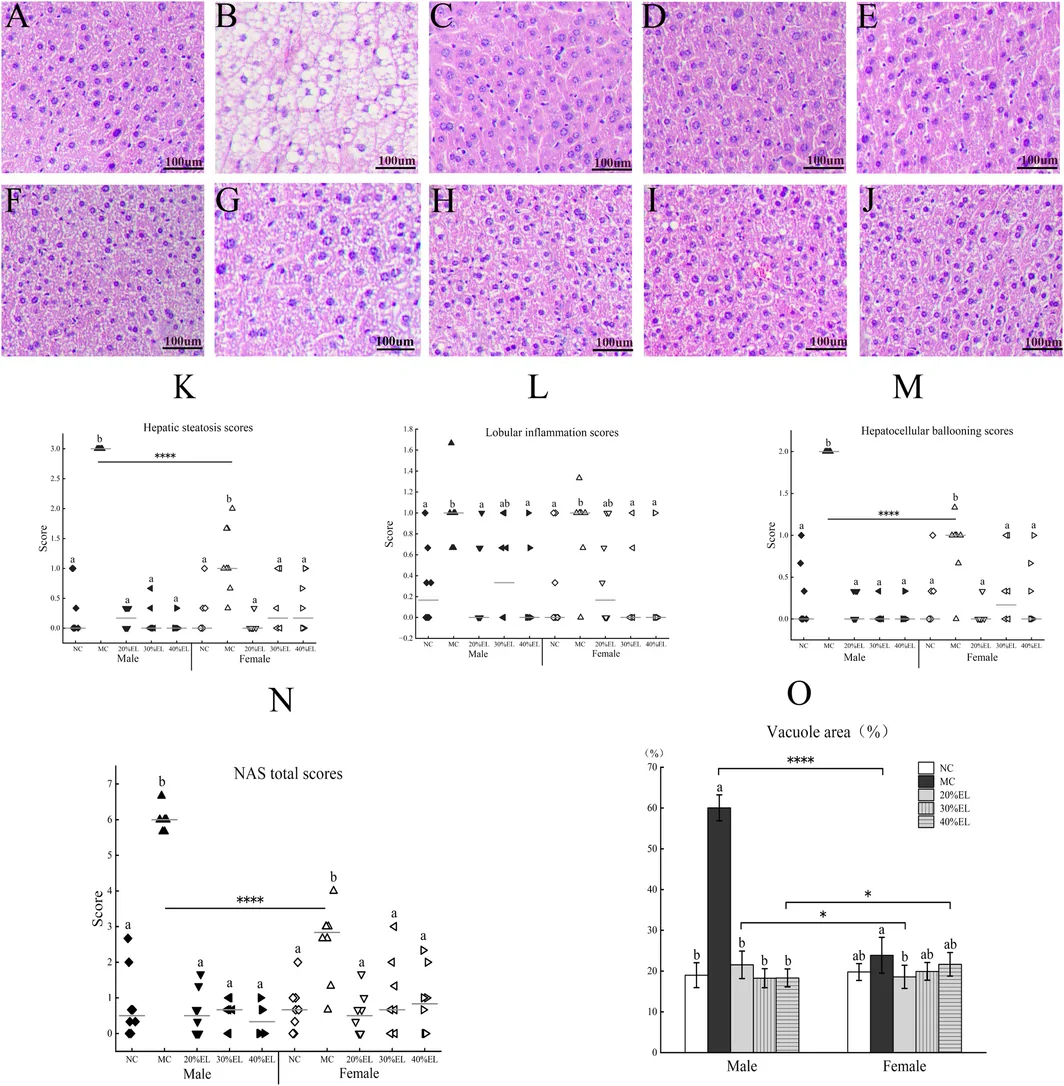
Liver histology and pathological scoring in mice. (A–E) Representative H&E‐stained liver sections from male mice in the NC, MC, 20% EL, 30% EL, and 40% EL groups, respectively. (F–J) Representative H&E‐stained liver sections from female mice in the NC, MC, 20% EL, 30% EL, and 40% EL groups, respectively. Scale bar: 100 μm (A–J). (K–N) Quantitative statistical results of hepatic steatosis score (K), inflammation score (L), ballooning score (M), and total NAS score (N) for each group. Data in (K–N) are skewed and are presented with gray horizontal lines indicating the median, and individual data points representing individual mouse scores, *n* = 8. (O) Quantitative statistical results of the percentage of hepatic vacuolar area for each group. Data are presented as mean ± SD, *n* = 24. Within the same sex, different letters indicate statistically significant differences among groups; asterisks denote significant differences between male and female mice under the same intervention (*p < 0.05; **p < 0.01; ***p < 0.001; ****p < 0.0001).

H&E staining revealed marked hepatic steatosis in the male MC group (Figure 4B), which was substantially attenuated in the 20%, 30% and 40% EL groups (Figure 4C–E). No appreciable steatosis was observed in any female group (Figure 4F–J). To quantify hepatic histological injury, the NAS components for steatosis, inflammation and ballooning were assessed in all groupsSteatosis and ballooning scores were significantly higher in both male and female MC mice than in all other groups, and both scores were significantly higher in MC males than in MC females (Figure 4K,M; *p* < 0.0001). Total NAS (Figure 4N) was significantly higher in MC mice (males, 6.00 ± 0.64; females, 2.54 ± 1.05) than in the corresponding EL groups, and was significantly higher in males than in females (*p* < 0.0001). Using the clinical threshold of NAS ≥ 5, male MC mice (mean score > 5) met the histological criteria for NAFLD. Moreover, the percentage area of hepatic vacuoles was significantly greater in male MC mice than in all other groups and than in female MC mice (Figure 4O,P < 0.0001), consistent with the increased microvesicular lipid droplets seen on H&E staining.

### 3.4 Hepatic Lipid Content and Key Enzymes

**FIGURE 5:**
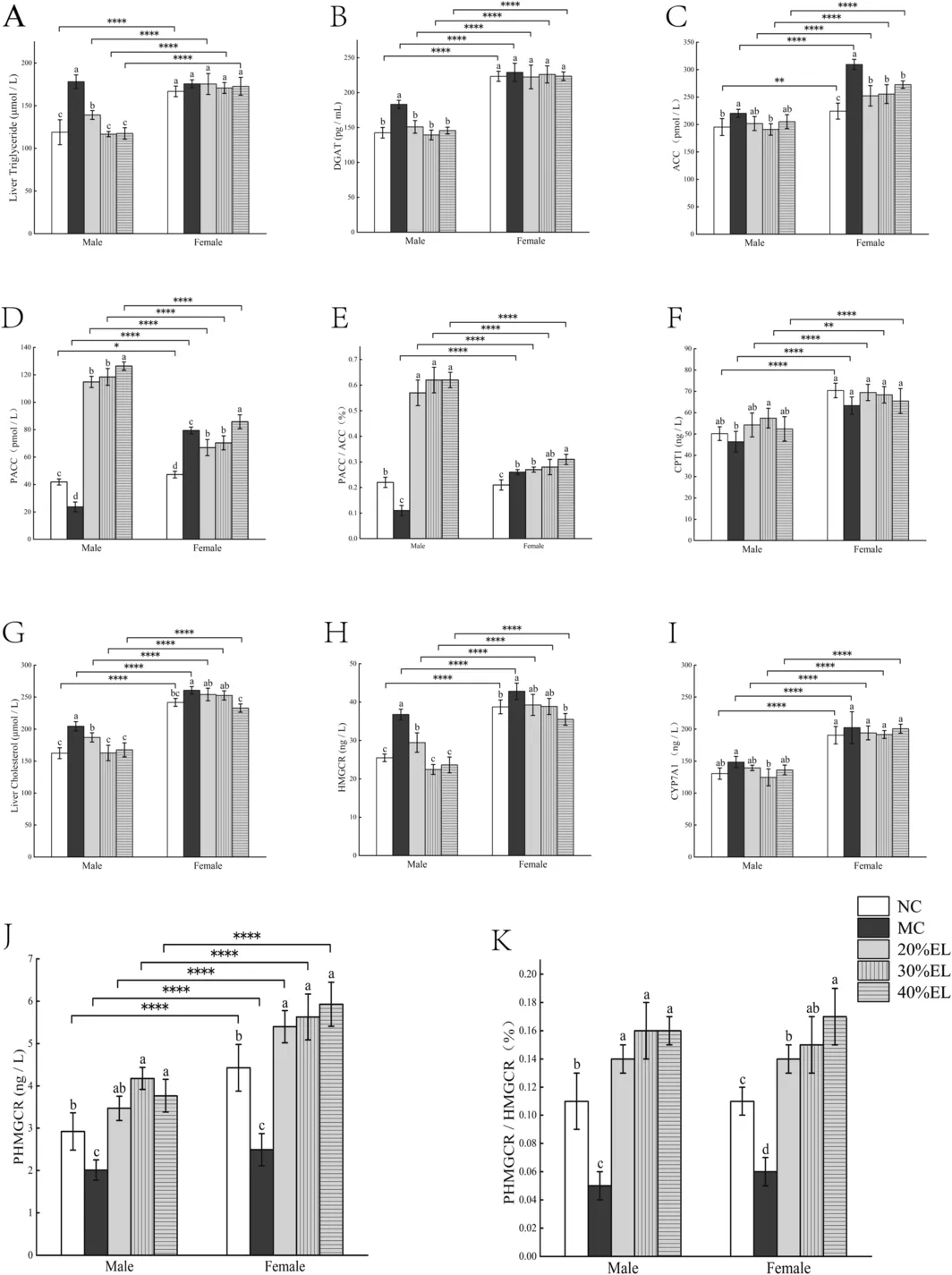
Hepatic lipid content and key enzymes in mice. (A) Hepatic TG content. (B) Hepatic DGAT content. (C) Hepatic ACC content. (D) Hepatic PACC content. (E) PACC/ACC ratio. (F) Hepatic CPT1 content. (G) Hepatic TC content. (H) Hepatic HMGCR content. (I) Hepatic CYP7A1 content. (J) Hepatic PHMGCR content. (K) PHMGCR/HMGCR ratio. Data are presented as mean ± SEM (*n* = 8). Within the same sex, different letters indicate statistically significant differences among groups; asterisks denote significant differences between male and female mice under the same intervention (*p < 0.05; **p < 0.01; ***p < 0.001; ****p < 0.0001).

Relative to the NC group, male MC mice showed significant increases in both hepatic TG and TC contents, whereas female MC mice showed a significant increase only in TC (*p* < 0.05) and no appreciable change in TG. Compared with the MC group, hepatic TG and TC were significantly reduced in all three male EL groups; values in the 30% and 40% EL groups were comparable to those of the NC group. In female mice, a significant reduction in TC was observed only with 40% EL (*p* < 0.05), while the other EL groups remained unchanged (Figure 5A,G). Key enzymes of hepatic TG and TC metabolism were next assessed (Figure 5B–F,H–K). Relative to the NC group, male MC mice exhibited significantly higher DGAT content and significantly lower pACC/ACC and pHMGCR/HMGCR ratios (*p* < 0.05). Female MC mice showed a significantly higher pACC/ACC ratio and a significantly lower pHMGCR/HMGCR ratio (*p* < 0.05). No significant changes in CPT1 or CYP7A1 were detected in either sex (*p* > 0.05). Compared with the MC group, pACC/ACC and pHMGCR/HMGCR ratios increased significantly in all EL groups in both sexes (*p* < 0.05); the greatest increases were observed at 30% EL in males and at 40% EL in females. In males, DGAT content was significantly lower in all three EL groups (*p* < 0.05). Significant changes in CPT1 and CYP7A1 were observed only in the male 30% EL group; no differences were detected in the other male EL groups or in any female EL group (*p* < 0.05).

### 3.5 Hepatic Metabolomics

#### 3.5.1 Data Quality Assessment

**FIGURE 6:**
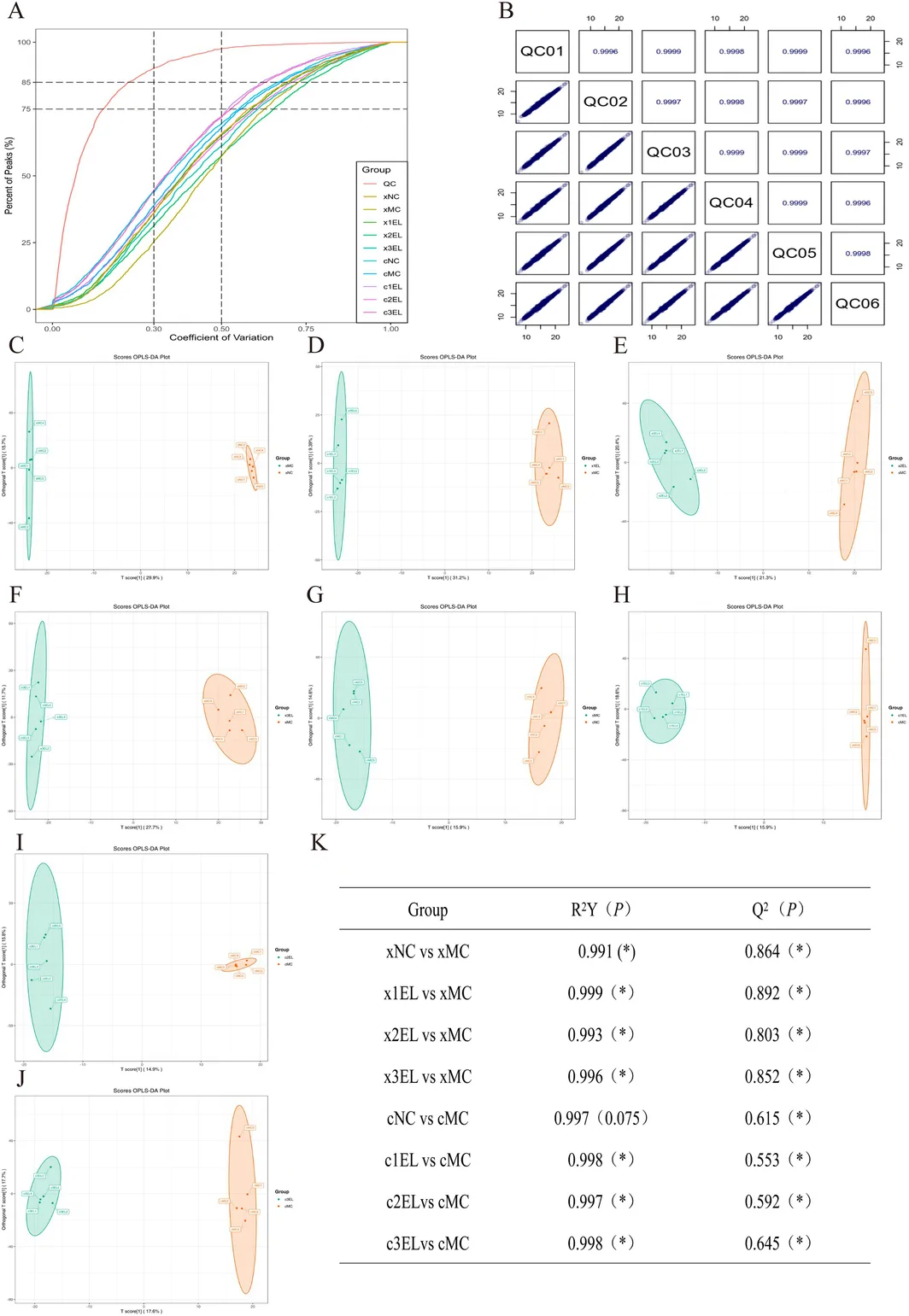
Metabolomic data quality assessment. (A) Distribution of coefficients of CV for each sample group; QC, quality control samples. (B) Correlation matrix of QC samples. (C–F) OPLS‐DA score plots for male comparisons: NC versus MC, 20% EL versus MC, 30% EL versus MC, and 40% EL versus MC, respectively. (G–J) OPLS‐DA score plots for female comparisons: NC versus MC, 20% EL versus MC, 30% EL versus MC, and 40% EL versus MC, respectively. (K) OPLS‐DA model validation parameters. *R* ^2^ *Y* represents the proportion of variance of the *Y* matrix explained by the model, and *Q*_2_ indicates the predictive ability of the model. *p < 0.05. *n* = 5. Abbreviations for group designations in the figure: c1EL, female 20% EL group; c2EL, female 30% EL group; c3EL, female 40% EL group; cMC, female MC group; cNC, female NC group; x1EL, male 20% EL group; x2EL, male 30% EL group; x3EL, male 40% EL group; xMC, male MC group; xNC, male NC group.

Quality control analysis showed that > 85% of metabolites had a coefficient of variation (CV) < 0.5, and > 75% had a CV < 0.3. Pearson correlation coefficients were all near 1, indicating high reproducibility (Figure 6A,B). Prior to supervised analysis, unsupervised PCA was performed on all samples. PCA score plots (Figure S1) showed distinct, non‐overlapping metabolic profiles for all groups, except for partial overlap between the female 30% EL and MC groups, pointing to the presence of differential metabolites. OPLS‐DA—a supervised method that maximizes class separation—revealed clear discrimination among groups (Figure 6C–J), confirming distinct metabolic profiles. Model validity was assessed with a 200‐permutation test. *R* ^2^ *Y* and *Q* ^2^ values are presented in Figure 6K, and the permutation plots are shown in Figure S2. Models were considered reliable when *Q* ^2^ > 0.5 and *p* < 0.05 (Triba et al. 2015); *R* ^2^ *Y* near 1 indicated good fit. As the permutation test for cNC vs. cMC gave *p* > 0.05 for *R* ^2^ *Y*—indicating a risk of overfitting—this pairwise comparison was excluded from further analysis.

#### 3.5.2 Differential Metabolite Identification

**FIGURE 7:**
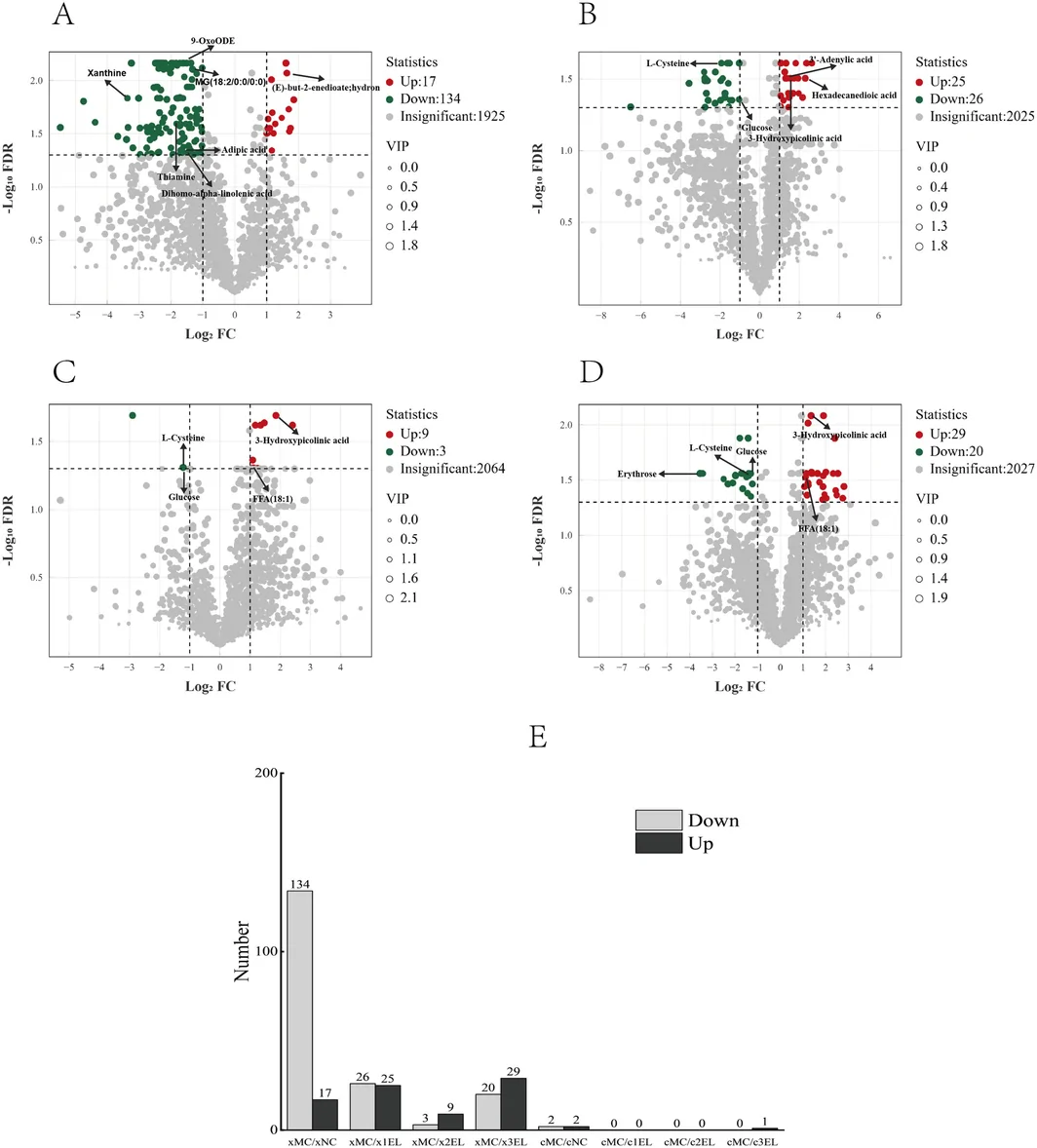
Differential hepatic metabolites in mice. (A–D) Volcano plots of differentially abundant metabolites in the indicated male comparisons: NC versus MC, 20% EL versus MC, 30% EL versus MC, and 40% EL versus MC. (E) Summary of differential metabolites across all comparisons in male and female mice.

After excluding the female MC and NC groups and applying the screening thresholds (VIP > 1, |log_2_(FC)| > 1, FDR < 0.05), significantly altered metabolites were detected only in comparisons involving the four male groups (NC, 20% EL, 30% EL, and 40% EL versus MC; Figure 7A–D). Volcano plots revealed pronounced metabolic perturbations in the male groups, with striking differences in the numbers of up‐ and down‐regulated metabolites in each EL group relative to the MC group (Figure 7E).

#### 3.5.3 High‐Confidence Differential Metabolites

To increase identification reliability, we applied a secondary quality‐control filter requiring Level 1a structural confidence and a matching score > 0.8. Environmental contaminants and exogenous compounds identified by database matching (e.g., Trolox, isophorone, and thioguanine) were also excluded. After this filtering, a set of high‐confidence differential metabolites was retained for further analysis (Table S4).

### 3.6 Gut Microbiota Analysis

**FIGURE 8:**
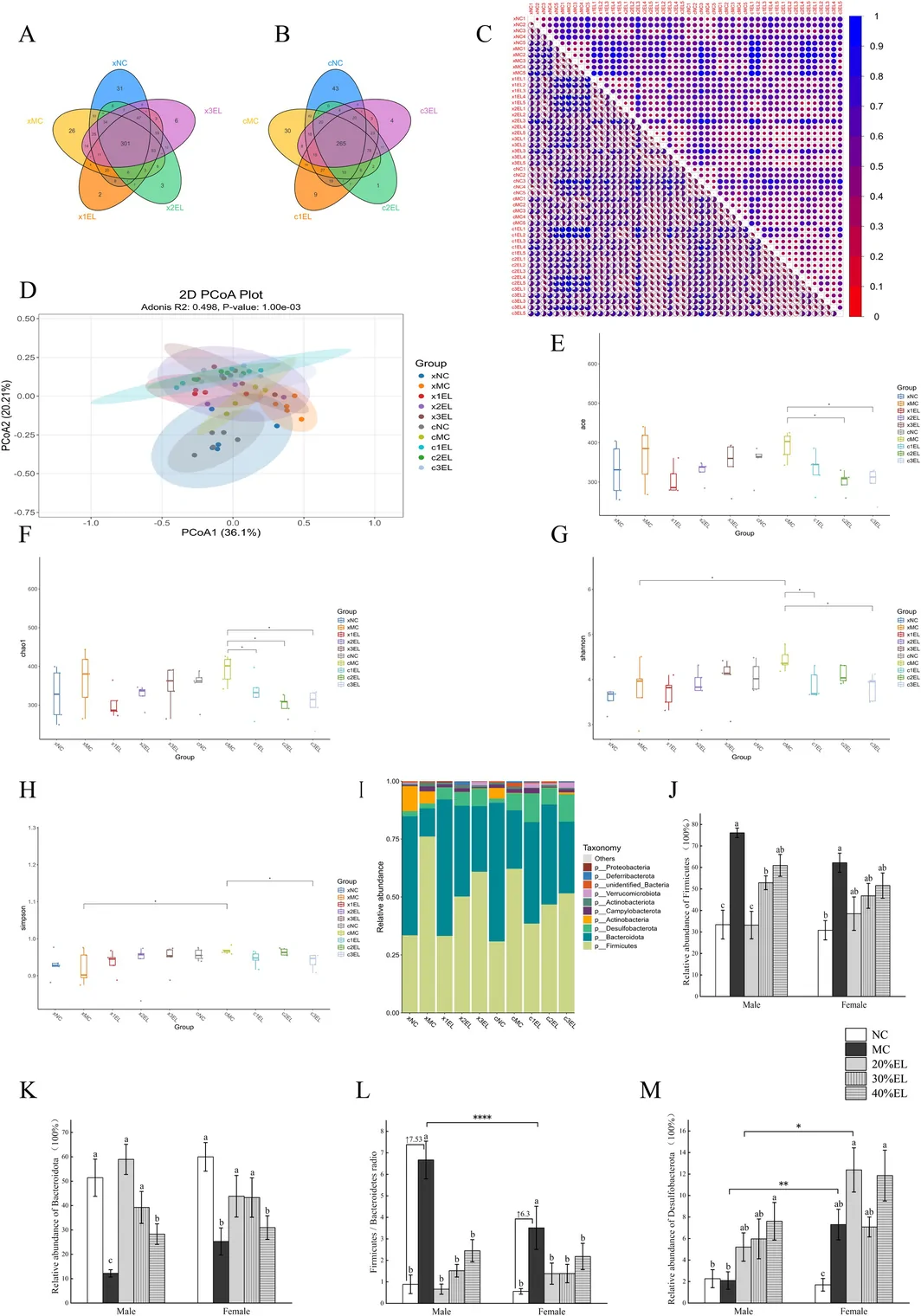
Gut microbiota analysis of mice (A) Venn diagram of ASVs in male mice. (B) Venn diagram of ASVs in female mice. (C) Heatmap of weighted UniFrac distances among samples; color gradient indicates community similarity (red, high similarity; blue, and high dissimilarity). (D) PCoA based on weighted UniFrac distance, with ellipses denoting group confidence intervals. The global ADONIS result is indicated above. (E–H) Box‐plots of α‐diversity indices: (E) Ace, (F) Chao1, (G) Shannon, (H) Simpson. (I) Stacked bar chart of phylum‐level relative abundance. (J) Firmicutes relative abundance. (K) Bacteroidota relative abundance. (L) Firmicutes/Bacteroidota (F/B) ratio. (M) Desulfobacterota relative abundance. Data are presented as mean ± SEM (*n* = 5). Within the same sex, different letters indicate statistically significant differences among groups; asterisks denote significant differences between male and female mice under the same intervention (*p < 0.05; **p < 0.01; ***p < 0.001; ****p < 0.0001). Abbreviations for group designations in the figure: c1EL, female 20% EL group; c2EL, female 30% EL group; c3EL, female 40% EL group; cMC, female MC group; cNC, female NC group; x1EL, male 20% EL group; x2EL, male 30% EL group; x3EL, male 40% EL group; xMC, male MC group; xNC, male NC group.

ASV Venn diagrams (Figure 8A,B) revealed 756 ASVs in male samples (301 shared by all groups) and 751 in female samples (265 shared). PCoA (Figure 8D) showed distinct clustering by group. The first two axes accounted for 56.31% of total variation. Global ADONIS testing attributed 49.8% of community variation to group identity (*R* ^2^ = 0.498, *p* = 0.001). A distance heatmap (Figure 8C) confirmed smaller within‐group distances (red) and larger between‐group distances (blue–purple). Complete pairwise ADONIS and ANOSIM results are provided in Appendix S3. No significant differences in community structure were found among EL groups of the same sex (male: x1EL vs. x2EL, x2EL vs. x3EL; female: c1EL vs. c2EL, c1EL vs. c3EL, c2EL vs. c3EL; all *p* > 0.05). All other pairwise comparisons were significantly different (*p* < 0.05).

α‐diversity indices (Ace, Chao1, Shannon and Simpson; Figure 8E–H) did not differ significantly in any male EL group relative to the MC group. In females, however, all four indices were significantly reduced in the 40% EL group (*p* < 0.05). Firmicutes, Bacteroidota and Desulfobacterota were the dominant gut phyla (Figure 8I). Compared with the NC group, MC mice of both sexes showed significantly higher Firmicutes and lower Bacteroidota abundances, leading to a marked increase in the Firmicutes/Bacteroidota (F/B) ratio (all *p* < 0.05). The F/B ratio was significantly higher in male MC mice (7.53) than in female MC mice (6.30). Relative to the MC group, 20% EL males had significantly lower Firmicutes abundance (*p* < 0.05), which gradually recovered with increasing EL intensity. In females, Firmicutes abundance tended to increase across EL groups but did not differ significantly from the MC group (*p* > 0.05). Desulfobacterota abundance in the MC group also displayed a pronounced sex‐dependent difference. Female MC mice had significantly higher abundance than the NC group (*p* < 0.05), whereas male MC mice showed no change (*p* > 0.05). Relative to the MC group, Desulfobacterota increased significantly only in the 40% EL group in males, but in all three EL groups in females (*p* < 0.05).

## 4 Discussion

Overweight/obesity can induce hepatic lipid metabolic disturbances; left untreated, this can progress from simple steatotic liver disease to metabolic dysfunction‐associated steatohepatitis, hepatic fibrosis and cirrhosis (Canfora et al. 2019). IR drives hepatic lipid accumulation by promoting fatty acid synthesis, inhibiting fatty acid oxidation, and activating inflammatory/oxidative stress pathways (Samuel and Shulman 2018). Moreover, IR stimulates hepatic secretion of very‐low‐density lipoprotein (VLDL) (van Zwol et al. 2024), which transports hepatic TG and TC into the circulation and is then converted to LDL through a series of enzymatic steps (van Zwol et al. 2024).

In this study, HFD‐induced overweight/obese mice developed IR that was more severe in males than in females. Relative to the NC group, male MC mice exhibited marked hepatic TG and TC accumulation and elevated plasma TG, TC and LDL levels, consistent with their IR; in contrast, female MC mice showed no significant change in hepatic TG content, which was inconsistent with the IR pattern. Elevated serum E2 in female MC mice may partly account for the lack of marked hepatic lipid alterations by promoting estrogen receptor α‐mediated hepatic TG and TC assembly and secretion, thereby maintaining basal intrahepatic lipid levels (Palmisano et al. 2017). This may explain the unchanged hepatic TG but markedly elevated serum TG in female MC mice. Moreover, estrogen preferentially promotes subcutaneous fat storage, whereas androgens favor visceral fat accumulation (Steiner and Berry 2022; Tchernof et al. 2018). This may partly explain the greater proportional increase in subcutaneous fat in female MC mice relative to males.

Our findings demonstrate that short‐term EL with normal protein intake markedly ameliorates insulin resistance, sex hormone dysregulation, and other obesity‐associated metabolic disturbances in both male and female overweight/obese mice. Moreover, short‐term EL with normal protein intake effectively reversed aberrant hepatic TG accumulation in overweight/obese male mice, as confirmed by liver histology. However, the hepatic lipid metabolic response to graded EL was strongly sex‐dependent: in males, EL markedly reduced hepatic TG and TC to near‐NC levels, with the greatest effect at 30% EL; in females, only 40% EL significantly reduced hepatic TC. Combined with white adipose tissue findings, under short‐term (1‐week) EL, females may preferentially mobilize stored white adipose tissue for energy, leaving hepatic TG metabolism relatively unaffected.

To elucidate the mechanisms underlying these sex‐ and group‐specific differences, we assessed key enzymes governing hepatic TG and TC anabolism and catabolism. Hepatic TG synthesis is primarily mediated by DGAT and ACC; TG catabolism depends largely on CPT1, which shuttles fatty acids into mitochondria for β‐oxidation (Wang et al. 2015). Hepatic TC metabolism is jointly regulated by HMGCR, the rate‐limiting enzyme for synthesis, and CYP7A1, the rate‐limiting enzyme for catabolism (Wang et al. 2015). The activities of ACC and HMGCR are primarily governed by phosphorylation; increased pACC/ACC and pHMGCR/HMGCR ratios signify inactivation of these enzymes and consequently suppress hepatic TG and TC synthesis (Steinberg and Kemp 2009).

In male mice, all EL groups showed significantly lower DGAT protein levels and reduced ACC and HMGCR activities relative to the MC group, with the greatest changes at 30% EL, indicating marked suppression of TG/TC synthesis. Catabolic enzyme changes were confined to the 30% EL group, where CPT1 was upregulated and CYP7A1 was downregulated; although CYP7A1 downregulation is unfavorable for cholesterol catabolism, the net reduction in hepatic TC suggests that inhibition of synthesis was dominant, likely explaining the greatest improvement at 30% EL in males. In females, HMGCR activity was lowest in the 40% EL group, leading to maximal inhibition of cholesterol synthesis, which may account for the optimal improvement at 40% EL.

To explore EL‐induced hepatic lipid metabolic changes, we performed untargeted liver metabolomics, which enables broad, unbiased detection of small‐molecule metabolites and related pathways (Nicholson et al. 2002). However, due to inherent limitations in coverage and identification accuracy, stringent filtering (VIP > 1, |log_2_(fold change)| > 1, FDR < 0.05) retained only a limited set of differential metabolites, and pathway enrichment analysis was not significant (Barupal and Fiehn 2017). We therefore discuss only individual metabolites.

Under short‐term EL with normal protein intake, hepatic metabolomic responses were highly sex‐specific. In males, hepatic glucose declined in all EL groups alongside blood glucose, indicating that EL accelerated hepatic glucose consumption beyond the replenishment capacity of gluconeogenesis. Moreover, adaptive changes occurred in lipid pathways. One such change involves L‐cysteine, a thiol‐based reductant that maintains HMGCR in its active conformation through thiol–disulfide exchange, thereby protecting the enzyme from oxidative damage and degradation (Belcastro et al. 2017; Hang et al. 2021). Downregulation of L‐cysteine in all male EL groups may attenuate this protective mechanism, reducing HMGCR content and activity, cholesterol synthesis, and TC production, consistent with the observed decreases in hepatic TC and HMGCR. Elevated oleic acid, hexadecanedioic acid, and 3‐hydroxypicolinic acid in all male EL groups may reflect enhanced hepatic TG catabolism, consistent with lower hepatic TG. In addition to these shared changes, 3′‐AMP was specifically upregulated at 20% EL relative to the MC group. 3′‐AMP, an intermediate of RNA degradation and coenzyme A catabolism (Verrier et al. 2013), is not directly involved in lipid regulation, but its elevation indirectly indicates enhanced hepatic catabolic activity under energy restriction. In the 40% EL group, erythrose was specifically downregulated; as an overflow product of the pentose phosphate pathway, its reduction may reflect decreased pathway flux and NADPH supply, suppressing hepatic reductive anabolism (Fuentes‐Lemus et al. 2023).

In marked contrast to males, female mice showed no significant hepatic metabolomic alterations relative to the MC group despite comparable weight loss, consistent with unchanged hepatic lipid levels in energy‐restricted females and underscoring a sex‐specific hepatic response to overweight/obesity.

In the normal mouse gut microbiota, Firmicutes and Bacteroidota are the dominant phyla, together accounting for > 90% of the community (Canfora et al. 2019). In this study, HFD‐induced overweight/obese mice of both sexes consistently showed higher Firmicutes abundance, lower Bacteroidota abundance and a markedly elevated Firmicutes/Bacteroidota (F/B) ratio. Although an elevated F/B ratio is often considered a hallmark of obesity reflecting enhanced energy‐harvesting capacity (Abeltino et al. 2024; Liu et al. 2025), its association with obesity remains controversial, as other studies have reported no increase or the opposite trend (Politi et al. 2023). Thus, the F/B ratio alone should not be used to define obesity‐associated gut dysbiosis, as it varies substantially with dietary composition and energy intake (Magne et al. 2020). In the present study, the F/B ratio was significantly elevated in overweight/obese mice of both sexes, supporting that HFD‐induced overweight/obesity drives this shift. Compared with the MC group, all EL groups had markedly lower energy intake and higher protein/carbohydrate intakes; the F/B ratio declined relative to MC but trended upward across increasing EL levels as energy and carbohydrate intake decreased progressively. These observations suggest complex, opposing effects of energy and macronutrient intakes on Firmicutes and Bacteroidota, though mechanisms warrant further study.

In this study, Desulfobacterota was the third most abundant phylum in the mouse gut. The response of Desulfobacterota to HFD‐induced obesity remains controversial, with sex‐dependent discrepancies reported. Lefebvre et al. (Lefebvre et al. 2024) described a significant increase in both sexes, whereas Zhu et al. (Zhu et al. 2023) found an increase only in obese males, with no change in females. In contrast, only female MC mice showed a significant elevation in our study, with no change in males, likely reflecting differences in HFD duration, dietary composition, and obesity severity. Short‐term EL with normal protein intake also significantly increased Desulfobacterota abundance in most mice, especially females, suggesting disturbed intestinal sulfur metabolism homeostasis, although the biological relevance remains unclear. However, since significant microbiota changes were detected only at the phylum level, specific bacterial taxa responsive to EL or linked to hepatic lipid metabolism could not be identified.

In summary, HFD‐induced overweight/obesity disrupted hepatic lipid metabolism and gut microbiota in mice, with more pronounced alterations in males. Short‐term EL with normal protein intake attenuated these disturbances by modulating hepatic TG/cholesterol synthesis and catabolism, with greater improvements in males, and reduced the gut Firmicutes/Bacteroidota ratio in overweight/obese mice.

This study has several limitations. First, C57BL/6J mice exhibit variable weight‐gain propensity on a high‐fat diet, and some are diet‐resistant (Enriori et al. 2007); consequently, only 80% of HFD‐fed mice became overweight/obese, and the limited number prevented stratification into overweight/obese subgroups. Second, because the HFD and the EL diets differed in macronutrient composition, benefits should be interpreted as combined effects of caloric restriction and altered carbohydrate‐to‐fat ratio under consistent protein intake, rather than caloric restriction alone. Third, using NC mice as the reference may confound direct EL effects with secondary consequences of weight loss. Fourth, we did not synchronize female oestrous cycles, introducing within‐group variation in estrogen that may confound results. Finally, budget constraints limited omics analyses to five randomly selected mice per group; despite randomization and FDR correction, reduced sample size may have limited statistical power to detect subtle differences.

## Author Contributions

**Ying Tian:** investigation, conceptualization, writing – review and editing, writing – original draft, funding acquisition, supervision, data curation, resources. **Yao Tang:** investigation, methodology, writing – original draft, software, formal analysis, visualization. **Mingxia Gu:** investigation, funding acquisition, methodology, validation, supervision. **Jiawei Gong:** supervision, project administration, validation, software. **Ying Zhou:** investigation, methodology. **Yuqing He:** investigation, methodology. **Keyu Huang:** investigation, methodology.

## Funding

The authors have nothing to report.

## Ethics Statement

All procedures and animal care in this study were approved by the Yangzhou University Laboratory Animal Ethics Committee (No. 202407030) and conducted in accordance with the ARRIVE guidelines and the Guide for the Care and Use of Laboratory Animals published by the National Institutes of Health.

## Conflicts of Interest

The authors declare no conflicts of interest.

## Supporting information


**Figure S1:** PCA.

**Figure S2:** Verification of the OPLS‐DA model.

**Appendix S1:** Detailed information on all metabolites.

**Appendix S2:** Phylum‐level taxonomic information of all samples.

**Appendix S3:** Complete pairwise ADONIS and ANOSIM results.

**Table S1:** Composition of the diets.
**Table S2:** Gradient conditions of the mobile phase for the T3 chromatographic column.
**Table S3:** *p* values and effect sizes of main effects and interactions from two‐way ANOVA and linear mixed models (LMM).
**Table S4:** Differential metabolite profiles across distinct classes in male groups.

## Data Availability

The datasets generated and/or analyzed during the current study are available in the GSA and OMIX database at the NGDC under accession numbers CRA048007 and OMIX019437, respectively. They can be accessed via https://ngdc.cncb.ac.cn/gsa/s/76CE85wC and https://ngdc.cncb.ac.cn/omix/preview/KjyMwkqdgp.
