# Supplementary material for: Effects of Short‐Term Energy Limitation at Different Levels With Normal Protein Intake on Hepatic Lipid Metabolism and the Gut Microbiota in Overweight/Obese Mice
Source: Food Sci Nutr. 2026 Sep 14;14(9):e72362. doi: 10.1002/fsn3.72362 (PMC13575506; doi:10.1002/fsn3.72362)
Supplement: Supplementary file 1 [file FSN3-14-e72362-s004.docx]

**Table S1.** Composition of the diets

| Category | Carbohydrate  (g/100g) | Fat  (g/100g) | Protein  (g/100g) | **Energy**  (kcal/100g) |
| --- | --- | --- | --- | --- |
| Standard diets | 20 | 5 | 60 | 365 |
| High-fat diets | 20 | 35 | 32 | 523 |
| 20%EL diets | 25 | 5 | 55 | 365 |
| 30%EL diets | 29 | 5 | 51 | 365 |
| 40%EL diets | 33 | 5 | 47 | 365 |

**Table S2.** Gradient conditions of the mobile phase for the T3 chromatographic column

| Time(min) | A (%) | B (%) |
| --- | --- | --- |
| 0.0 | 95 | 5 |
| 2.0 | 80 | 20 |
| 5.0 | 40 | 60 |
| 6.0 | 1 | 99 |
| 7.5 | 1 | 99 |
| 7.6 | 95 | 5 |
| 10.0 | 95 | 5 |

**Table S3.** P-values and effect sizes of main effects and interactions from two-way ANOVA and linear mixed models (LMM).

| Dependent variable | Main effect 1 (P) | Main effect 2 (P) | Interaction (P) | Normality (P) | Homogeneity (P) | Statistical method |
| --- | --- | --- | --- | --- | --- | --- |
| Male BW (0–9 wks) | Diet(0.001) | Week(0.0001) | Diet * Week (0.0001) | See Note | **_** | Linear Mixed Model ( LMM) |
| Male BW (9–11 wks) | Diet(0.01) | Week(0.0001) | Diet * Week (0.001) | See Note | **_** | LMM |
| Female BW (0–11 wks) | Diet(0.0001) | Week(0.0001) | Diet * Week (0.001) | See Note | **_** | LMM |
| Female BW (11–12 wks) | Diet(0.01) | Week(0.0001) | Diet * Week (0.034) | See Note | **_** | LMM |
| Food intake | Sex(0.0001) | Group(0.0001) | Sex * Group (0.0001) | See Note | 0.48 | Two-way ANOVA |
| Energy intake | Sex(0.0001) | Group(0.0001) | Sex * Group (0.642) | See Note | 0.095 | Two-way ANOVA |
| CHO intake | Sex(0.0001) | Group(0.0001) | Sex * Group (0.103) | See Note | 0.283 | Two-way ANOVA |
| PRO intake | Sex(0.0001) | Group(0.002) | Sex * Group (0.394) | See Note | 0.674 | Two-way ANOVA |
| FAT intake | Sex(0.001) | Group(0.019) | Sext * Group (0.906) | See Note | 0.0001 | Robust two-way ANOVA (trimmed means) |
| WAT weight | Sex(0.0001) | Group(0.0001) | Sex * Group (0.049) | See Note | 0.055 | Two-way ANOVA |
| Blood glucose | Sex(0.845) | Group(0.0001) | Sex * Group (0.0001) | See Note | 0.095 | Two-way ANOVA |
| Insulin | Sex(0.0001) | Group(0.0001) | Sex * Group (0.017) | See Note | 0.224 | Two-way ANOVA |
| Glucagon | Sex(0.058) | Group(0.0001) | Sex * Group (0.0001) | See Note | 0.771 | Two-way ANOVA |
| IR | Sex(0.0015) | Group(0.0001) | Sex * Group (0.0001) | See Note | 0.068 | Two-way ANOVA |
| Serum TG | Sex(0.0016) | Group(0.0001) | Sex * Group (0.002) | See Note | 0.319 | Two-way ANOVA |
| Serum TC | Sex(0.0001) | Group(0.0001) | Sex * Group (0.0001) | See Note | 0.099 | Two-way ANOVA |
| LDL | Sex(0.979) | Group(0.0001) | Sex * Group (0.005) | See Note | 0.379 | Two-way ANOVA |
| HDL | Sex(0.0001) | Group(0.0001) | Sex * Group (0.0001) | See Note | 0.092 | Two-way ANOVA |
| T | Sex(0.002) | Group(0.001) | Sex * Group (0.017) | See Note | 0.0001 | Robust two-way ANOVA (trimmed means) |
| E2 | Sex(0.039) | Group(0.001) | Sex * Group (0.049) | See Note | 0.0001 | Robust two-way ANOVA (trimmed means) |
| Steatosis (0–3) | Sex(0.001) | Group(0.0001) | Sex * Group (0.0001) | See Note | 0.056 | Robust two-way ANOVA (trimmed means) |
| Inflammation (0–3) | Sex(0.221) | Group(0.006) | Sex * Group (0.273) | See Note | 0.848 | Robust two-way ANOVA (trimmed means) |
| Ballooning (0–2) | Sex(0.019) | Group(0.0001) | Sex * Group (0.0001) | See Note | 0.361 | Robust two-way ANOVA (trimmed means) |
| Total NAS (0–8) | Sex(0.0001) | Group(0.0001) | Sex * Group (0.0001) | See Note | 0.284 | Two-way ANOVA |
| Vacuolation area (%) | Sex(0.0001) | Group(0.0001) | Sex * Group (0.0001) | See Note | 0.86 | Two-way ANOVA |
| Hepatic TG | Sex(0.001) | Group(0.001) | Sex * Group (0.001) | See Note | 0.009 | Robust two-way ANOVA (trimmed means) |
| DGAT | Sex(0.0001) | Group(0.0001) | Sex * Group (0.0001) | See Note | 0.051 | Two-way ANOVA |
| ACC | Sex(0.0001) | Group(0.0001) | Sex * Group (0.0001) | See Note | 0.45 | Two-way ANOVA |
| CPT1 | Sex(0.0001) | Group(0.0001) | Sex * Group (0.049) | See Note | 0.651 | Two-way ANOVA |
| Hepatic TC | Sex(0.0001) | Group(0.0001) | Sex * Group (0.0001) | See Note | 0.377 | Two-way ANOVA |
| HMGCR | Sex(0.0001) | Group(0.0001) | Sex * Group (0.0001) | See Note | 0.743 | Two-way ANOVA |
| CYP7A1 | Sex(0.013) | Group(0.001) | Sex * Group (0.0385) | See Note | 0.001 | Robust two-way ANOVA (trimmed means) |
| PACC | Sex(0.0001) | Group(0.0001) | Sex * Group (0.0001) | See Note | 0.068 | Two-way ANOVA |
| PACC/ACC | Sex(0.0001) | Group(0.0001) | Sex * Group (0.0001) | See Note | 0.018 | Robust two-way ANOVA (trimmed means) |
| PHMGCR | Sex(0.0001) | Group(0.0001) | Sex * Group (0.0001) | See Note | 0.528 | Two-way ANOVA |
| PHMGCR/HMGCR | Sex(0.934) | Group(0.0001) | Sex * Group (0.305) | See Note | 0.387 | Two-way ANOVA |
| Firmicutes | Sex(0.19) | Group(0.0001) | Sex * Group (0.626) | See Note | 0.71 | Two-way ANOVA |
| Bacteroidota | Sex(0.549) | Group(0.0001) | Sex * Group (0.321) | See Note | 0.77 | Two-way ANOVA |
| F/B | Sex(0.084) | Group(0.0001) | Sex * Group (0.017) | See Note | 0.053 | Two-way ANOVA |
| Desulfobacterota | Sex(0.007) | Group(0.002) | Sex * Group (0.047) | See Note | 0.759 | Two-way ANOVA |

Note：Normality was assessed using the Shapiro-Wilk test and P-P plots; detailed results are presented in the supplementary figure S3 and S4.

**Table S4.**Differential metabolite profiles across distinct classes in male groups.

| Group | | Metabolite Class | | | | |
| --- | --- | --- | --- | --- | --- | --- |
|  |  | Lipids | Amino acids / Peptides | Nucleotides / Purines | Carbohydrates | Others |
| Male | xNC VS xMC | MAG18:2↓  Dihomo-alpha-linolenic acid↓  9-OxoODE↓ | _ | Xanthine↓ | _ | Adipic acid↓  (E)-but-2-enedioate;hydron ↑  Thiamine↓ |
|  | xMC VS x1EL | Hexadecanedioic acid↑ | L-Cysteine↓ | 3'-Adenylic acid↑ | Glucose↓ | 3-Hydroxypicolinic acid↑ |
|  | xMC VS x2EL | FFA(18:1)↑ | L-Cysteine↓ | _ | Glucose↓ | 3-Hydroxypicolinic acid↑ |
|  | xMC VS x3EL | FFA(18:1)↑ | L-Cysteine↓ | _ | Glucose↓  Erythrose↓ | 3-Hydroxypicolinic acid↑ |
